# “*Fine synergies*” describe motor adaptation in people with drop foot in a way that supplements traditional “*coarse synergies*”

**DOI:** 10.3389/fspor.2023.1080170

**Published:** 2023-02-17

**Authors:** Angelo Bartsch-Jimenez, Michalina Błażkiewicz, Hesam Azadjou, Ryan Novotny, Francisco J. Valero-Cuevas

**Affiliations:** ^1^ Division of Biokinesiology and Physical Therapy, University of Southern California, Los Angeles, CA, United States; ^2^ Józef Piłsudski University of Physical Education in Warsaw, Warsaw, Poland; ^3^ Biomedical Engineering Department, University of Southern California, Los Angeles, CA, United States; ^4^ Neuroscience Graduate Program, University of Southern California, Los Angeles, CA, United States; ^5^ Escuela de Kinesiología, Facultad de Medicina, Universidad de Valparaíso, Valparaíso, Chile

**Keywords:** electromyography, muscle synergies, non-negative matrix factorization, drop foot, gait

## Abstract

Synergy analysis via dimensionality reduction is a standard approach in biomechanics to capture the dominant features of limb kinematics or muscle activation signals, which can be called “coarse synergies.” Here we demonstrate that the less dominant features of these signals, which are often explicitly disregarded or considered noise, can nevertheless exhibit “fine synergies” that reveal subtle, yet functionally important, adaptations. To find the coarse synergies, we applied non-negative matrix factorization (NMF) to unilateral EMG data from eight muscles of the involved leg in ten people with drop-foot (DF), and of the right leg of 16 unimpaired (control) participants. We then extracted the fine synergies for each group by removing the coarse synergies (i.e., first two factors explaining ≥85% of variance) from the data and applying Principal Component Analysis (PCA) to those residuals. Surprisingly, the time histories and structure of the coarse EMG synergies showed few differences between DF and controls—even though the kinematics of drop-foot gait is evidently different from unimpaired gait. In contrast, the structure of the fine EMG synergies (as per their PCA loadings) showed significant differences between groups. In particular, loadings for *Tibialis Anterior*, *Peroneus Longus*, *Gastrocnemius Lateralis*, *Biceps* and *Rectus Femoris*, *Vastus Medialis* and *Lateralis* muscles differed between groups (p<0.05). We conclude that the multiple differences found in the structure of the fine synergies extracted from EMG in people with drop-foot vs. unimpaired controls—not visible in the coarse synergies—likely reflect differences in their motor strategies. Coarse synergies, in contrast, seem to mostly reflect the gross features of EMG in bipedal gait that must be met by all participants—and thus show few differences between groups. However, drawing insights into the clinical origin of these differences requires well-controlled clinical trials. We propose that fine synergies should not be disregarded in biomechanical analysis, as they may be more informative of the disruption and adaptation of muscle coordination strategies in participants due to drop-foot, age and/or other gait impairments.

## 1. Introduction

Applying dimensionality reduction techniques to kinematic or electromyographic (EMG) data is a form of unsupervised learning (1, 2) to capture the lower-dimensional structure of the neural control of movement (1, 3–8). Independently on whether or not these “synergies” are of neural origin (4, 7), they are “descriptive” (8, 9) (in a mathematical sense) of the basis functions that best explain a high percentage of the variance in the data.^1^ The investigator must first determine *a priori* if linear or nonlinear basis functions are most appropriate, and what is the discrete number of basis functions (i.e., synergies) that explain a “high enough” percentage of the variance (1). In practice, methods that produce linear basis functions are most popular such as Non-Negative Matrix Factorization (NMF) (5, 10), Principal Component Analysis (PCA) (11), Independent Component Analysis (ICA) (12), and Factor Analysis (FA) (13).

In the fields of biomechanics and neuromechanics, the number of synergies that together explain 80- 90% of the variance are considered sufficient to explain the dominant characteristics of the data and, therefore, most informative (3–8, 14, 15). We call these “*coarse synergies*.” The residuals from the coarse synergies (i.e., which represent the remaining 20–10% of the variance) are, by construction, data (i) in which the investigator is *a priori* not interested (because they explicitly set the cut-off for variance explained), (ii) which cannot be accounted for by the linear model (a by-product of the preferred method (1)), or (iii) are considered noise (an assumption which must be proven) (16, 17). In either case, they are considered irrelevant or unimportant.

Here, we question this traditional interpretation of coarse synergies and the assumptions about their residuals to explore the subtle ways in which synergies can differ across populations. Our rationale is that there are coarse mechanical features of, in this case, locomotion that must be common to all participants—and are therefore not very informative of differences across populations. Therefore, we look to residuals as a more informative source of subtle differences.

In particular, here we focus on analysing the residuals after removing coarse synergies to establish whether or not they are irrelevant, and if they are informative of fine features of muscle coordination that are not captured by the coarse synergies. To do so, we apply dimensionality reduction to the residuals of the coarse synergies to extract “*fine synergies*.” As a first example of this approach, we use EMG from leg muscles during locomotion to compare coarse and fine synergies between people with drop foot (DF) vs. unimpaired control participants (C).

## 2. Materials and methods

### 2.1. Participants

Two groups of people participated in this study. Ten individuals with clinically diagnosed unilateral drop foot without comorbidities that prevented locomotion formed the experimental group (DF). Their mean age was 52.9±17.9 years, height 174.8±9.1 cm, and body mass 68.8±18.7 kg. The following medical diagnosis were represented: peroneal nerve palsy secondary to lumbar disc herniation (n=2); post motor vehicle injury (n=1); progressive muscular dystrophy (n=3); surgical removal of a tumor at the level of the head of the fibula (n=2); ischemic disease of the lower limbs surgically fitted with stents (n=1); and, amyotrophic lateral sclerosis (n=1). In daily life, all participants were ambulatory and did not report dependence on a wheelchair. During test day, they verbally declared a good health and physical condition to participate in the study. Sixteen unimpaired participants with a mean age of 25.3±7.1 years, height of 176.6±6.8 cm and body mass of 74.1±10.5 kg constituted the control group (C). All participants gave their informed written consent to participate in this study. The procedures were approved by the Ethical Committee of the Medical Center of Postgraduate Education in Warsaw, Poland (84/PB/2016).

### 2.2. Instrumentation and data collection

Unilateral surface EMG (sEMG) was collected from eight muscles using a Noraxon system (Noraxon USA. Inc., USA). Data were collected from the involved limb of persons from the DF group, and from the right limb from control participants. The activity was recorded from the following eight muscles: *Tensor Fasciae Latae* (TFL), *Biceps Femoris* (BF), *Peroneus Longus* (PL), *Gastrocnemius Lateralis* (GL), *Vastus Lateralis* (VL), *Tibialis Anterior* (TA), *Vastus Medialis* (VM) and *Rectus Femoris* (RF). For each participant, the bipolar Ag–AgCl EMG electrodes (10-mm diameter, 20-mm dipole distance) location was identified according to guidelines for electrode placement developed by the Surface Electromyography for the Non-Invasive Assessment of Muscles (SENIAM) project and verified based on clinical muscle tests.

All participants walked barefoot and naturally at their self-selected speed along a 10m walkway. Trials with incidents were discarded from further analysis and the procedure was repeated. Two force plates (Kistler Holding AG, Switzerland) were used to determine ground reaction forces using Nexus 1.7.1 software, which afterwards was confirmed manually for each participant. Data was then exported to the Vicon Polygon system, which independently divided the gait into individual cycles and calculated the gait spatio-temporal parameters. EMG and Force plate systems were synchronized and had a sampling frequency of 1000 Hz. After data collection from the Drop foot group, kinetic and kinematic data were visually inspected to determine the results’ homogeneity (Supplementary Figure 6).

### 2.3. Data analysis and muscle synergy extraction

Surface EMG signals were high-pass filtered to remove movement artifacts, using a third-order Butterworth high-pass filter at 20 Hz. On-line sEMG signals were displayed for inspection of the signal quality during measurement. The sEMG signals were rectified and smoothed with a 2 Hz second-order Butterworth low-pass filter to obtain the muscle contraction linear envelope. The third gait cycle from each participant was selected for analysis based on ground reaction forces data. The sEMG envelopes were processed into a time normalized sEMG profile (i.e., from 0 to 100% of gait cycle, starting at heel strike). Next, each muscle’s sEMG time series for each participant was normalized by the maximal peak value demonstrated by that specific muscle across gait cycles. Therefore, the magnitude of muscle activity was not taken into consideration in this temporal analysis.

Extraction of coarse synergies: We used the NMF algorithm to extract muscle synergies and their corresponding activation coefficients (i.e., weights) (10). This method calculates a set of synergy weights ($W_{m\times n}$) and synergy activations ($A_{x\times j}$), such that sEMG=W×A+residuals, where n is the number of synergies, m is the number of muscles (eight in this study), and j is equal to the number of sEMG data points (15). The residuals are defined as the difference between the experimental sEMG envelopes and the sEMG envelopes reconstructed from the product of the synergy weights and activations. The procedure to select the number of coarse synergies was to include as many as necessary to have ≥80% of variance accounted for (VAF) (15). To compare the coarse features of muscle coordination between control (C) and drop foot (DF) groups, we applied Statistical Parametric Mapping (SPM) to the reconstructed activity profiles, and a mixed design robust ANOVA with trimmed means (18) to compare the muscle weights extracted from the two coarse synergies that accounted for ≥80% of variance. The *spm1d* package (www.spm1d.org) was used to perform SPM analysis (19). SPM was used to compare the reconstructed muscles activity profiles between groups C and DF to detect whether the coarse synergies showed statistically significant differences over the gait cycle.

Extraction of fine synergies: To extract the residual sEMG signals, the above reconstructed signals were subtracted from the original experimental sEMG envelopes. PCA was applied to the residual components of EMG to extract the fine synergies for each participant in both groups. In contrast to the experimental sEMG envelopes that have a 0 floor and 1 ceiling—which NMF can accommodate best—the residuals are zero-mean time-series for which PCA is appropriate. For each participant, we extracted the principal components (PC’s) and their loadings, which were then normalized based on the highest loading per participant for both groups (20).

To compare the fine features of muscle coordination between control (C) and drop foot (DF) groups, we also applied Statistical Parametric Mapping (SPM) to the reconstructed activity profiles, and a mixed design robust ANOVA with trimmed means to compare the normalized muscle loadings extracted from the fine synergies. Non parametric post-hoc analyses were used to compare individual muscle pairs when the results from the robust ANOVA revealed a main or interaction effect. All statistical procedures were performed with RStudio (RStudio Team, MA, USA).

## 3. Results

### 3.1. Spatio-temporal parameters

The spatiotemporal parameters of both groups are listed in Table 1, and were compared using t-tests for independent samples. Cadence for the DF group was 81.1±2.42 steps/min, while the Control group was 90.6±4.45 steps/min. Step length was 0.5±0.07 m for the DF group and 0.66±0.09 m for the Control group. Step width was 0.11±0.02 for both groups. Stride time was 1.43±0.14 (s) for DF and 1.29±0.07 (s) for the Control group. Finally, walking speed was 0.8±0.03 (m/s) for the DF group and 1.33±0.06 (m/s) for the Control group. All spatiotemporal parameters were significantly different between groups (p<0.01), except for Step Width (Table 1).

**Table 1:** Spatiotemporal gait patterns mean (± standard deviation) in drop foot (DF) and Control (C) groups.

	DF group	C group	*p*-Value
Cadence (steps/min)	81.11±2.42	90.65±4.45	0.0001
Step length (m)	0.5±0.07	0.66±0.09	0.0002
Step width (m)	0.11±0.02	0.11±0.02	—
Stride time (s)	1.43±0.14	1.29±0.07	0.0090
Walking speed (m/s)	0.8±0.03	1.33±0.06	0.0001

#### 3.1.1. Coarse synergies

**Figure 1:**
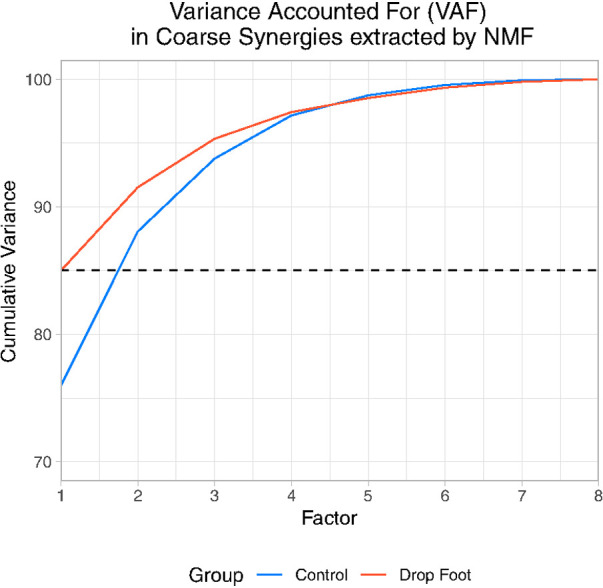
Cumulative variance accounted for each Factor extracted by NMF in drop foot (DF) and Control (C).

As expected, only two NMF factors sufficed to explain the gross features of muscle coordination in both groups (Supplementary Tables S2, S3). In the control group two factors explained an average of 88.1±3% of variance accounted for (Supplementary Table S2 and Figure 1). Whereas for the drop foot group, the first two factors explained, on average, 91.52 ±3.96% of variance accounted for (Supplementary Table S3 and Figure 1). We defined these first two factors that explain ≥85% to be the *coarse synergies* for the Control and Drop Foot groups.

**Figure 2:**
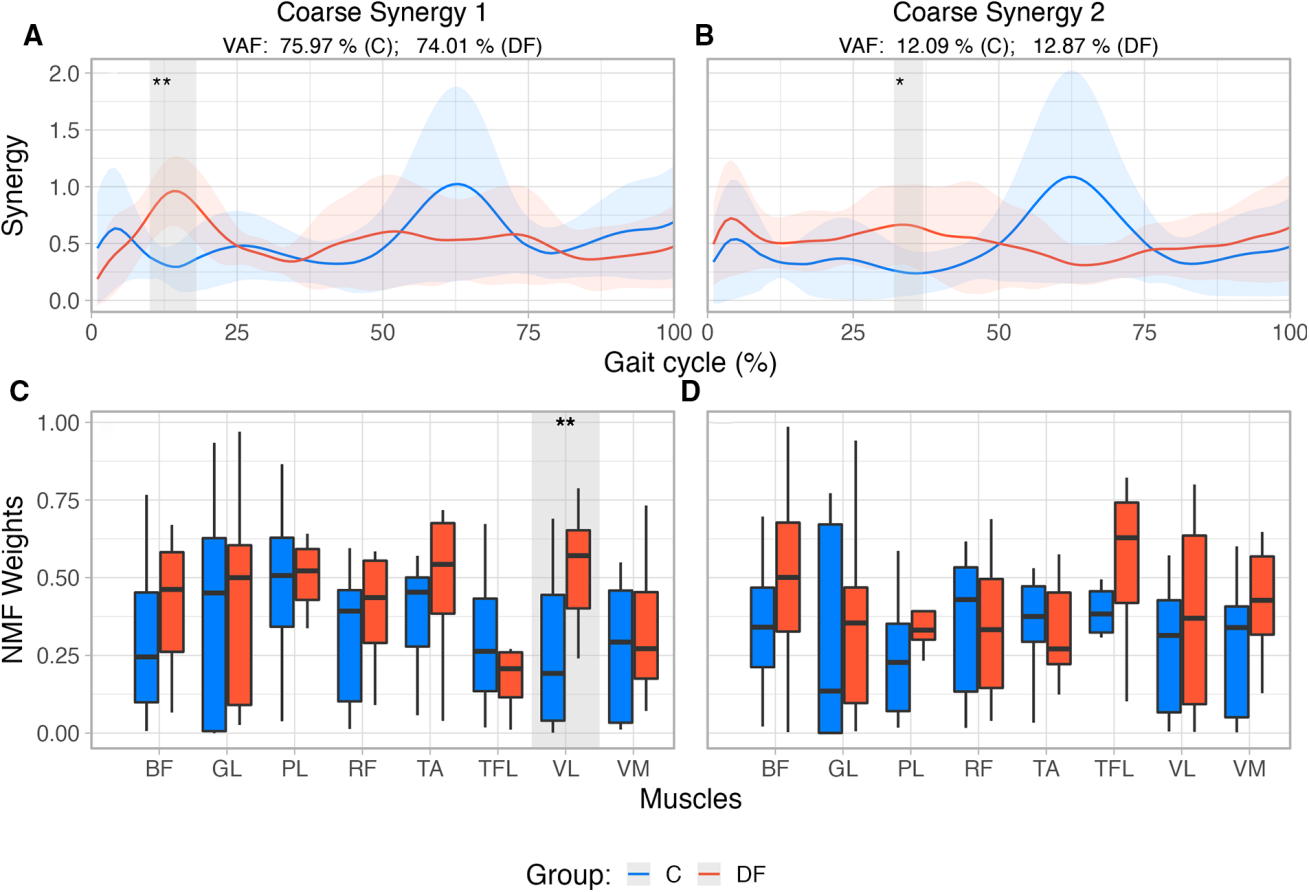
(**A**,**B**) Reconstructed muscle activity profiles based on weights extracted from first two *coarse synergies* for each group, accounting for >86% of variance in each group. Shaded areas identify differences between groups based on SPM{t} results and their corresponding levels of significance. (**C**,**D**) Coarse synergies muscle weights extracted from NMF for unimpaired control participants (C) and persons with drop foot (DF). ${}^{*}$Significant at 5%; ${}^{**}$Significant at 1%.

The time histories of the coarse EMG synergies in the DF group showed few differences compared to Controls. While there are visual differences between the DF and Control groups, the only statistically significant ones (as per SPM, p<0.01) occurred in the first *coarse synergy* from 10 to 18% of the gait cycle (Figure 2A). For the second *coarse synergy*, significant differences (p=0.015) were only observed from 32% to 37% of the gait cycle (Figure 2B).

The structure of the coarse EMG synergies showed differences only for one muscle between the DF and Control groups. Muscle weights^2^ extracted from NMF (Figures 2C,D) were compared using a Robust mixed effects ANOVA model. In the *first coarse* synergy, the analysis revealed a main effect for Muscle (p<0.01), and Group (p=0.032), with no interaction (Muscle×Group, p=0.3). Post-hoc analysis revealed significant differences between groups for muscle VL (p<0.01) only. Comparison of muscles weights extracted from the second coarse synergy did not show main effects for Muscle (p=0.07), Group (p=0.05) nor interaction (Muscle×Group, p=0.53).

#### 3.1.2. Fine synergies

**Figure 3:**
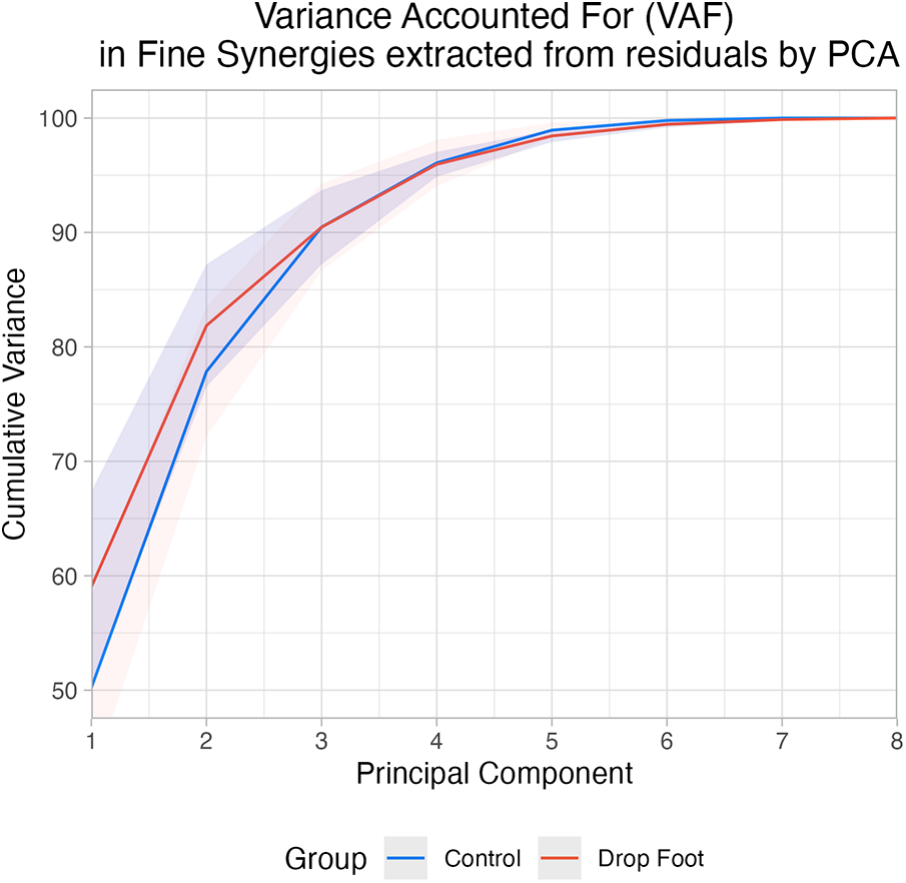
Cumulative variance accounted for each PC extracted from residuals by PCA in drop foot (DF) and control (C).

Three *fine synergies* sufficed to explain ≥85% of variance in the residuals in both groups: 90.47% (±3.79 SD) and 90.46% (±3.24 SD) in the Control and DF groups, respectively (Figure 3).

**Figure 4:**
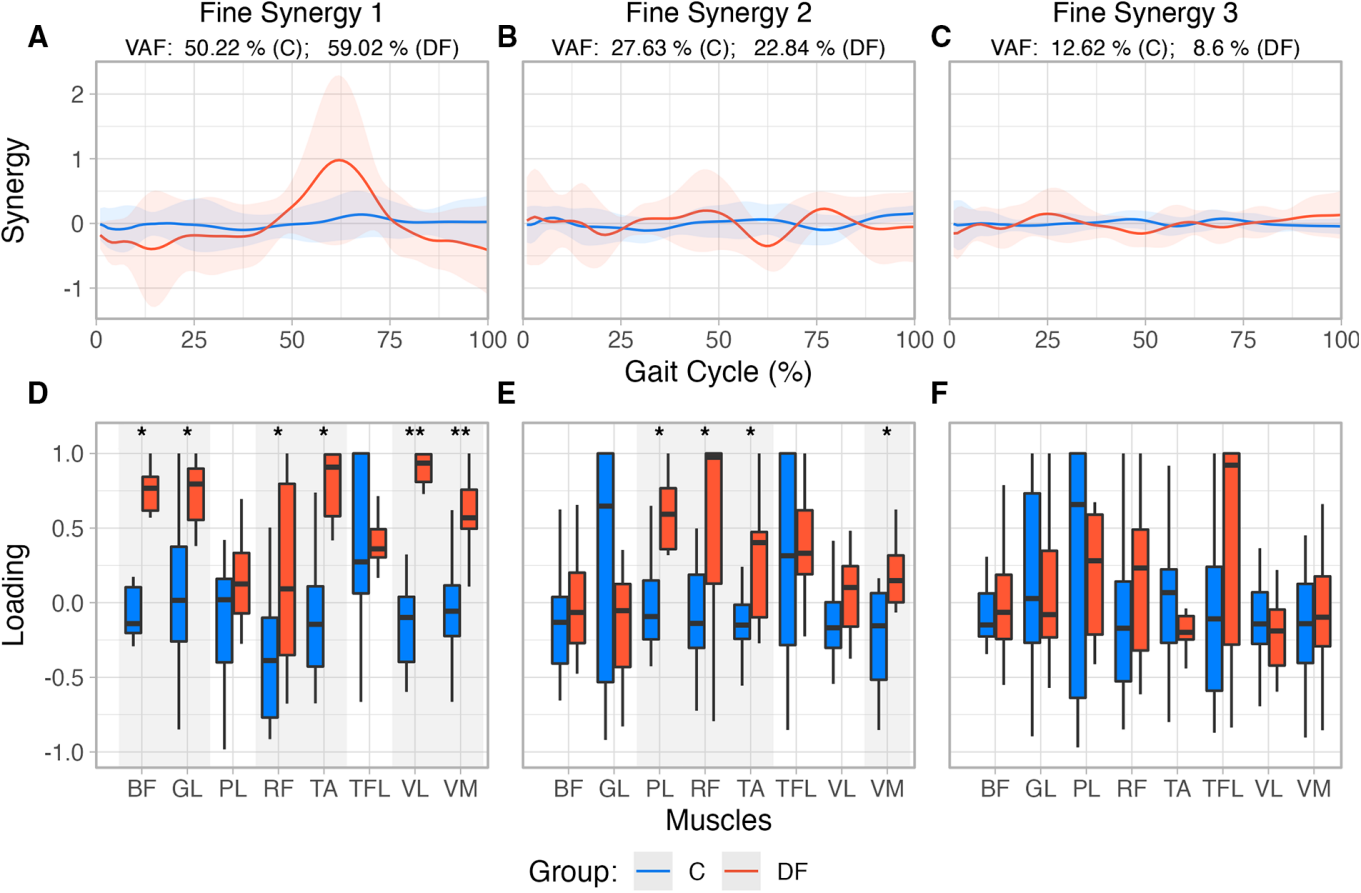
(**A**–**C**) Reconstructed muscle activity profiles based on loadings extracted from first three “fine synergies” for each group. (**D**–**F**) Fine synergies loadings extracted from PCA for unimpaired control participants and persons with drop foot. Also note the loadings in DF are in general closer to +1 in the DF case, indicating greater synergistic correlation among muscle activations.

SPM analysis did not reveal differences between groups at any level of significance in the histories of the three fine synergies (Figures 4A,C).

The structure of the first two fine synergies showed multiple statistically significant differences between the Control and DF groups, as per their loadings. Muscle loadings extracted from PCA (Figures 4D,F) were also compared using a Robust mixed effects ANOVA model, which revealed a main Group effect for the first and second *fine synergies* (p<0.01, for both synergies), and a Muscle main effect (p<0.01) in the second “fine synergy.” Post-hoc analysis revealed statistical differences between both groups for muscles TA (p=0.016), BF (p=0.038), RF (p=0.049), GL (p=0.015), VL (p=0.01) and VM (p=0.01) in the first synergy, and PL (p=0.024), RF (p=0.036), TA (p=0.031), and VM (p=0.036), in the second *fine synergy*.

The third fine synergy did not show differences in its structure between Control and DF groups. The third synergy did not have a main Muscle (p=0.40), Group (p=0.49), nor interaction effect (Muscle×Group, p=0.52). Moreover, all of their loadings tended to include or hover near zero. These results suggest the third fine synergy is likely unimportant to both groups (Figure 4F).

## 4. Discussion

Descriptive synergies which explain the majority of the variance in data (i.e., *coarse synergies*) are a common metric to compare performance across populations. We argue that coarse synergies, in the case of DF at least, can be uninformative about differences between groups as they mostly capture the dominant biomechanical features of locomotion common to all participants. We thus explored the notion that descriptive *fine synergies* extracted from the residuals to the *coarse synergies* may be—by virtue of containing subtler features—more informative of differences across populations.

Our results show this is the case when analyzing EMG signals from control and DF participants as the fine synergies showed the most differences across populations—potentially revealing subtle disruptions and adaptations of muscle coordination strategies in participants with DF.

An important methodological aspect of our approach is that we first used NMF on the EMG data, and then PCA on their residuals. Our rationale is twofold. NMF is a well-founded approach for analyzing rectified and normalized EMG signals that lie between values of 0 and 1 due to the non-negative input constraint to perform factorization. As such, it is better suited to extract coarse synergies (≥85% VAF) from processed EMG signals (10, 11, 21). The residuals of the EMG signals after removal of the coarse synergies are zero-mean by construction, and therefore PCA is the more appropriate technique for extracting fine synergies (1). We then focused on analyzing these residual EMG signals first and foremost to establish whether or not they had enough structure in their correlations to make them informative of fine features of muscle coordination that are not captured by the coarse synergies.

The nature of PCA loadings should be clarified before proceeding. PCA is a dimensionality reduction technique that approximates a high-dimensional signal with fewer basis vectors (PCs) that capture important features of correlations in the original signal. The values of PCA loadings have a range between −1 and 1, therefore, describe whether and how the elements of the original signal are correlated. Namely, loadings describe if there is structure to their correlations, or if their correlations hover near zero and therefore render the synergies informative. Importantly, PCA is obtained from the covariance matrix of the individual EMG signals, thus the correlations among EMG signals are what determine their loadings and not their overall level of activation. Therefore, a weakened muscle with a low level of activation—such as the TA in the DF group—can still have a loading close to 1 (or −1) in a PC if its activity is highly correlated (or anti-correlated) with the other muscles. On the other hand, a muscle could have a loading hovering near zero even if it is highly activated but uncorrelate with other muscles in that PC.

**Figure 5:**
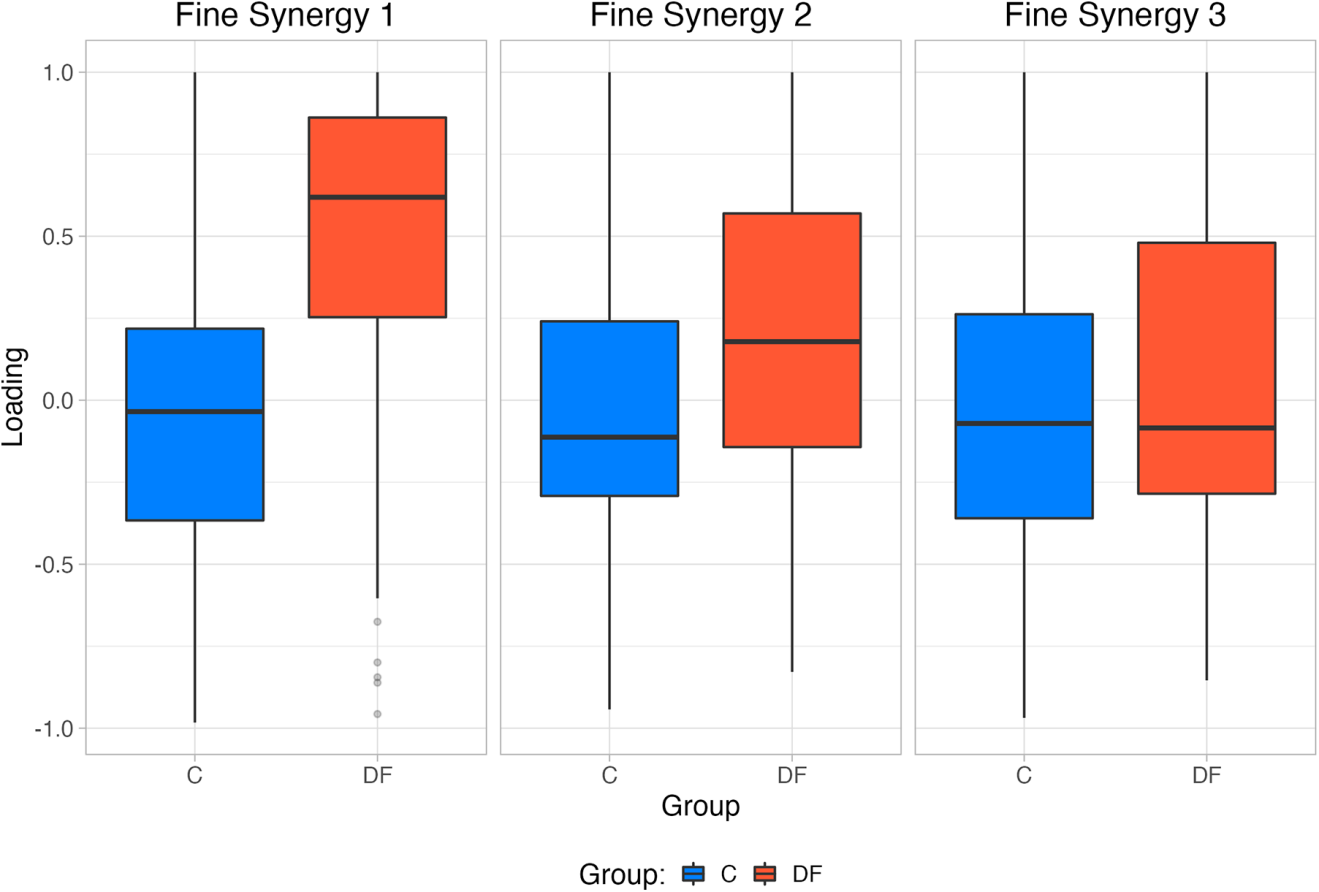
Mean Fine synergy loadings for each group extracted by PCA.

Given this preface, our results showed that the first two fine synergies in the DF participants were different from those in the controls. This is evidenced by the DF loadings being statistically different from controls in Figures 4, 5. This is also valid for TA—even though we know it is weaker in the DF group—because its loadings are statistically different in the first and second fine synergies compared to controls. In contrast, all three fine synergies of the control participants, and the third fine synergy of the DF group, show little correlation structure as they loadings are hovering near zero. Therefore, those fine synergies are uninformative.

Dimensionality reduction techniques to extract coarse synergies have known limitations (1, 4, 9, 16). For example, synergies are necessarily descriptive of the correlations among muscle activities; but do not necessarily speak to the actual neural control producing the task (9). In addition, PCA relies on signal normalization, which for EMG is performed via maximum voluntary contraction (MVC) of each muscle. However, this process is unreliable and true maximal force is difficult to attain in individuals with motor deficits (22). Here, we normalized EMG signals based on the maximal activity of each muscle during the gait cycle. We did this to prove that weakness based on changes in the EMG signal is not the only change in muscle activity between groups, and is actually a change in the correlation structure among muscles that produces differences between groups. Since we already know that the activity levels will be different across groups, by normalizing to the maximal activity during the gait cycle we make the amplitudes of the signals comparable to reveal differences in the correlations among muscle activations. If scaling down the signals due to weakness is the only change during DF, we should not have found differences in the muscle loadings compared to controls. The presence of these differences in the fine synergies and not in the coarse synergies highlights the ability of fine synergies to reveal compensatory motor coordination strategies.

In order to test the usefulness of coarse vs. fine synergies to detect differences across groups, we compared the DF group to the so-called clinically neurotypical group. We consider young self-declared unimpaired people as such. On the other hand, if we had considered an age-matched group to those with DF, we would have the concern that they might exhibit some comorbidities of aging that would confound our results. Initially, 15 older subjects were screened for enrollment in our study; however, they did not meet our inclusion criteria due to comorbidities. Thus we kept younger individuals as controls to avoid potential confounds of aging.

We recognize that our study had a small sample size, compared populations of different ages, and the number of electrodes may not fully capture the muscle activation patterns of the leg. However, to the best of our knowledge, aging does not affect kinetic and kinematic parameters during gait (23). While age could partly explain our results (or an interaction between age and DF), this could only be confirmed using a larger sample and a more complex experimental design that is beyond the scope of this work. Importantly, our goal was not to definitively declare DF from its various diagnoses, levels of impairment, clinical evolution (and/or age) as the main cause of differences between groups. We also do not claim that synergies of any kind can provide clinical insights unless and until they are used in the context of well-controlled clinical trials (which for DF is beyond the scope of this work). Rather, we used data from DF populations as a first example that allows us to question the traditional approach to, and interpretation of, descriptive ‘coarse synergies’ as biomarkers for changes in motor strategies. Our results show that changes due to DF (and/or aging) are not reflected in coarse synergies, further supporting the importance of analyzing “fine synergies”—which is the main topic and goal of our study.

To mitigate the limitations of our small sample size, we used robust inferential methods for hypothesis testing, which perform well with small sample sizes and when the assumptions of parametric statistics regarding normality and homoscedasticity are not met, and provide more accurate statistical results compared to classic parametric statistical techniques based on means comparisons (18).

From a technical perspective, our wired equipment limited the number of channels to record EMG signals from each participant to eight. We therefore chose to record the signals only from the affected side of each DF participant. Also, due to cable length, participants were only able to walk 10 m, the reason for which we analyze only the third cycle once they reached a stable gait pattern before starting to decelerate and come to a full stop. Therefore, we could not record EMG during three full strides at a participant’s comfortable speed to assess recording’s reliability (24).

Notwithstanding these limitations, we find that coarse synergies are not as informative of differences across populations during gait, as compared to fine synergies. In particular, we saw an increase in the correlation of the weakened TA muscle activation with other muscles in the DF group (i.e., higher loading value), which was also seen in most of the recorded muscles in the first and second fine synergies, with only *Tensor Fasciae Latae* not being statistically different in any synergy (Figure 4). In the DF group, the increased loading for the *Biceps Femoris* may act as a compensatory mechanism to decrease hip flexion during initial contact, potentially translating to a decreased step length. Additionally, the increased loading for the *Vastus Medialis* and *Lateralis* could represent a mechanism to decrease knee flexion during midstance. These changes have been previously reported in people with DF during ground clearance and foot-ground interaction (25). Previous findings have also shown that the presence of weakness during foot dorsiflexion in DF activates compensation strategies and influences muscle force and activation distribution (26). It was found that reduced forces of individual muscle groups of the ankle joint are compensated for by the increased strength of others acting on this joint (i.e. *Tibialis Posterior*, *Gastrocnemius Lateralis*), along with other muscles in neighboring joints (i.e. *Biceps Femoris*, *Rectus Femoris*, *Vastus Lateralis*, *Tensor Fasciae Latae*) (26). Considering that we found differences in PCA loadings within the same muscles (with the exception of *Tensor Fascia Latae*), our results from the fine synergies could reflect the same gait adaptations in the DF group as previously described.

Our results have allowed us to better characterize motor deficits and adaptations in persons with DF, based on differences in fine synergies as compared to control participants. This highlights the importance of considering not only the dominant features of a behavior (coarse synergies), but also the fine details revealed by fine synergies.

## Data Availability

The raw data supporting the conclusions of this article will be made available by the authors, without undue reservation.

## Footnotes

^1^That is, the original data can be approximated as a combination of the basis functions extracted from the original data.

^2^In NMF factors are described by their “weights,” whereas in PCA the term “loadings” is used.
